# Long-Term Outcome after Asphyxia and Therapeutic Hypothermia in Late Preterm Infants: A Pilot Study

**DOI:** 10.3390/healthcare9080994

**Published:** 2021-08-04

**Authors:** Hanne Lademann, Karl Abshagen, Anna Janning, Jan Däbritz, Dirk Olbertz

**Affiliations:** 1 Department of Pediatrics, Rostock University Medical Center, 18057 Rostock, Germany; hanne.lademann@med.uni-rostock.de; 2 Medical School, Rostock University Medical Center, 18055 Rostock, Germany; karl.abshagen@uni-rostock.de (K.A.); anna.janning@uni-rostock.de (A.J.); 3 Department of Neonatology and Neonatal Intensive Care, Südstadt Hospital Rostock, 18059 Rostock, Germany; dirk.olbertz@kliniksued-rostock.de

**Keywords:** very low birth weight, very preterm, hypoxic-ischemic encephalopathy, Bayley scales of infant development, cooling

## Abstract

Therapeutic hypothermia (THT) is the recommended treatment for neuroprotection in (near) term newborns that experience perinatal asphyxia with hypoxic-ischemic encephalopathy. The benefit of THT in preterm newborns is unknown. This pilot study aims to investigate long-term outcomes of late preterm asphyctic infants with and without THT compared to term infants. The single-center, retrospective analysis examined medical charts of infants with perinatal asphyxia born between 2008 and 2015. Long-term outcome was assessed using the Bayley Scales of Infant Development 2 at the age of (corrected) 24 months. Term (*n* = 31) and preterm (*n* = 8) infants with THT showed no differences regarding their long-term outcomes of psychomotor development (Psychomotor Developmental Index 101 ± 16 vs. 105 ± 11, *p* = 0.570), whereas preterm infants had a better mental outcome (Mental Developmental Index 105 ± 13 vs. 93 ± 18, *p* = 0.048). Preterm infants with and without (*n* = 69) THT showed a similar mental and psychomotor development (Mental Developmental Index 105 ± 13 vs. 96 ± 20, *p* = 0.527; Psychomotor Developmental Index 105 ± 11 vs. 105 ± 15, *p* = 0.927). The study highlights the importance of studying THT in asphyctic preterm infants. However, this study shows limitations and should not be used as a basis for decision-making in the clinical context. Results of a multicenter trial of THT for preterm infants (ID No.: CN-01540535) have to be awaited.

## 1. Introduction

Worldwide, nearly a quarter of neonatal deaths are related to perinatal asphyxia [1]. Perinatal asphyxia describes the oxygen deficiency of the fetus, due to a disturbed gas exchange between maternal and fetal blood, either intrauterine or during the birth process [2]. Hypoxic-ischemic encephalopathy (HIE) is a result of a severe asphyctic event resulting in a hypoperfusion by hypoxemic blood. Growth retardation, fever and infection increase the sensitivity of the brain to a hypoxic episode [3]. A lack of oxygen induces an anaerobic cell metabolism. The resulting acidosis, oxidative stress and deficiency of adenosine triphosphate (ATP) cause global depolarization [4,5]. The resulting accumulation of glutamate in the extracellular space leads to an influx and an ion imbalance and a cytotoxic edema. This process of necrosis is summarized as primary energy failure and can be accompanied by cerebral palsy. After primary energy failure, a short latent phase may be associated with the beginning of seizures, epilepsy and neurodevelopmental disabilities [3,6,7]. The secondary energy failure is shaped by apoptosis and lasts for 6–72 h [3]. In this phase, HIE develops [7].

Therapeutic hypothermia (THT) is considered to be the gold standard in the treatment of perinatal asphyxia to prevent HIE [8,9,10,11]. Due to the pathophysiology of HIE it has to be started during the first six hours after the hypoxic event [12,13]. A lower temperature results in a reduced production of reactive nitrogen and oxygen species with a reduction in associated damage [14]. Besides THT, other established neuroprotective measures include securing gas exchange, stabilizing circulation with catecholamines and optimizing the acid–base balance. For the improvement of the prognosis, the therapy of cerebral seizures and brain edema is indispensable [15].

The outcome of THT is strongly dependent on the severity of HIE. Newborns with mild HIE usually develop age-appropriately [16] and evidence is insufficient to recommend THT here [17]. In moderate and severe HIE, THT reduces mortality and morbidity [18,19]. Severe HIE without THT is almost invariably associated with permanent morbidity and has a mortality rate of 75% [20]. At 18 to 24 months, almost 100% of children with severe HIE have motor or cognitive deficits or spastic cerebral palsy. With THT, however, only 24% of these children show severe motor and cognitive developmental deficits. The incidence of spastic cerebral palsy is also reduced from 40% to 30% following THT in severe HIE [21].

To date, studies regarding THT have been limited to infants with a gestational age greater than 36 weeks. In asphyctic preterm infants there are no existing recommendations regarding THT [2]. Animal studies could figure out if cooling in preterm sheep models has a benefit [22].

Only two small studies have explicitly evaluated the short- and long-term outcome of THT in late preterm newborns, assuming that THT in preterm infants is feasible [23] but showing a “concerning” incidence of complications and combined outcome of death and neurodevelopmental impairment [24]. Two further studies included preterm infants greater than 35 gestational weeks but did not provide subgroup analysis of these patients [25,26]. The benefit of THT in preterm infants remains unclear [27,28,29]. Moreover, there seems to be a great heterogeneity regarding decision-making among neonatologists when it comes to the decision whether or not asphyctic preterm infants should be treated with THT [30].

Thus, the primary goal of this study was to evaluate mental and physical long-term outcome of late preterm newborn after perinatal asphyxia and THT. Secondly, differences and similarities between hospitalization and follow-up examination in both term and late preterm infants (with and without THT, respectively) shall be described.

## 2. Materials and Methods

### 2.1. Perinatal Asphyxia

In this single-center, retrospective but pilot study, infants, who have been encoded with the diagnosis “perinatal asphyxia” in the period 2008–2015 at the tertiary neonatal intensive care unit of the Südstadt Hospital Rostock, Germany, have been identified. Of these, infants fulfilling the definition of perinatal asphyxia according to national guidelines, which is herein defined as clinical signs of an HIE and umbilical artery pH (UapH) below 7.0 or a base deficit smaller than −16 mmol/L or a 5-min Apgar score below 6 points [2], have been included. Data of medical charts were summarized retrospectively with regard to baseline characteristics (gender, somatic data, birth data: UapH, base deficit, Apgar score). Infants with a gestational age <34 weeks were excluded, as therapeutic hypothermia has never been offered below this limit. Between 34 + 0 and 35 + 6 weeks of gestational age we offer THT as an individual treatment given all requirements were met. All parents gave their written consent to off-label use of THT in an informed consensus at birth of their infants. According to Azzopardi et al. [9] children with severe malformations or multi-morbidity have been excluded.

### 2.2. Hypoxic-Ischemic Encephalopathy

To characterize our HIE, cohort data of medical charts were summarized retrospectively according to the clinical phases of asphyxia and HIE. Therefore, clinical signs of encephalopathy (palsy, seizures, coma) were identified. In addition, the results of the amplitude-generated electroencephalograms (aEEG) have been considered. Pathological aEEG findings have been defined as burst suppression pattern, general amplitude slowing or spikes. Moreover, severity of HIE was classified by the scores of Sarnat and Sarnat [31].

### 2.3. Therapeutic Hypothermia

According to national guidelines [2] THT was performed in children with:

- Severe acidosis (pH ≤ 7.0 or a base deficit ≤ −16 mmol/L) in umbilical cord blood or a blood sample from the first hour of life.
- Clinical signs of a moderate or severe encephalopathy (severity 2 or 3 according to Sarnat and Sarnat [31] or pathological aEEG).
- Postnatal age ≤6 h.

According to national guidelines, exclusion criteria were severe congenital malformations, severe intracranial hemorrhages, multi-morbidity and conversely to the guidelines a gestational age <34 + 0, not <36 + 0 weeks [2].

THT was performed using a cooling blanket with a thermostat regulated fluid (TecoTherm NEO^®^ by Inspiration HealthCare, Leicester, United Kingdom). According to national guidelines a body temperature of 91.4–93.2 °F (33–34 °C) was reached within six hours after birth and maintained for 72 h [2]. Rewarming process was conducted with an increasing temperature of 32 °F (0.5 °C) per hour to a maximum of 99.5 °F (37.5 °C). Newborns without THT were placed in incubators with a target temperature of 98.6 °F (37 °C).

### 2.4. Outcome

Follow-up examination was performed at the age of (corrected) 24 months. Baseline characteristics (body weight, height, head circumference) were collected, as well as psychomotor and mental development assessed using Bayley Scales of Infant Development 2 (BSID 2). The BSID published by Nancy Bayley in 1969 and revised in 1993 [27] are known to be not only the most widely used method but also the gold standard in assessing neurodevelopment of infants born very preterm and with very low birth weight in the first 42 months of life [32,33]. BSID 2 was applied by trained physicians and resulted in two scores: the physical and mental development indices, composed of 138 and 178 items, respectively. In both scores, the mean value is 100 (standard deviation (SD) 15). Scores above 85 show regular performances; scores of 70–84 show a moderate; and scores below 70 show a severe delay of mental and psychomotor development [27]. Two groups of patients were considered (term/preterm) and divided into two subgroups each (with/without THT).

### 2.5. Statistics

Shapiro–Wilk test and graphic representation were performed to detect a normal distribution of our cohort regarding baseline characteristics. The chi-square test or Fisher’s exact probability was used to compare the nominal hospitalization data between children with and without THT and between term and preterm children with THT (seizures, palsy, coma, pathologic aEEG, HIE, death). To describe the central and distributional measurements we used mean value (mv) and standard deviation (SD) in all items except the Apgar score where we used median and interquartile range (IQR) as it is a more realistic notation. To compare quantitative variables, we used the Mann–Whitney U test (non-parametric variables e.g., results of the BSID 2) and the t-test (parametric variables, e.g., female, somatic parameters, base excess, UapH, Apgar score). To reduce the alpha error, we used the adjustment by Bonferroni–Holm in cases of pairwise comparison with more than two characteristics. *p*-values < 0.05 were found to be significant. Statistical analyses were performed by using IBM SPSS Statistics Version 22.0^®^.

### 2.6. Ethics

The study was approved by the Ethics Committee of the Medical Faculty of the University of Rostock, Germany (Approval No.: A 2020-0052).

## 3. Results

### 3.1. Perinatal Asphyxia

**Figure 1:**
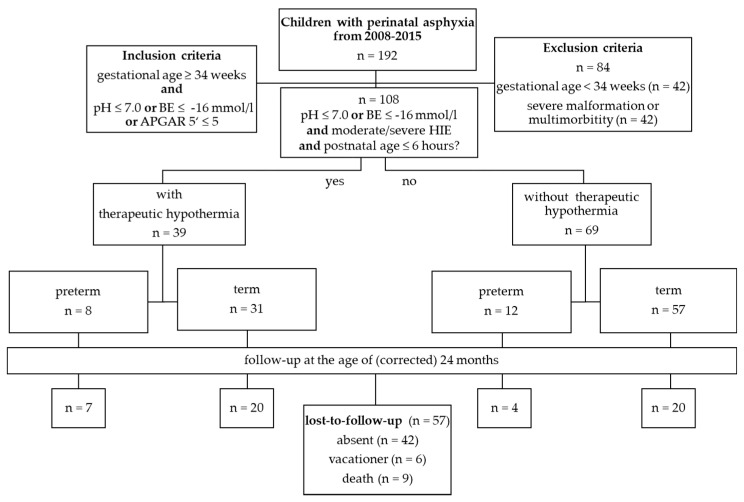
Overview of the inclusion and exclusion criteria of the study, therapeutic groups and follow-up-examinations. Overall, 192 children with perinatal asphyxia were identified between 2008 and 2015. A total of 109 infants met the inclusion criteria. A total of 39 infants showed clinical signs of a hypoxic-ischemic encephalopathy or correlating pathologic results in the amplitude-integrated electroencephalogram (aEEG) and were treated with THT. Eight of these infants were preterm newborns. Follow-up examinations were conducted at the age of (corrected) 24 months. Abbreviations: n—number, BE—Base excess, ‘—minute, HIE—hypoxic-ischemic encephalopathy.

The characteristics of the study population are shown in Figure 1. From a total of 20,890 births, a series of 192 infants was identified with the diagnosis “perinatal asphyxia”. Of these, 108 (0.5%) met the inclusion criteria. A total of 84 infants have been excluded because of an age under 34 gestational weeks (*n* = 42), malformations or multi-morbidity (*n* = 42). A total of 42 of 84 excluded children suffered from multi-morbidity (malformations of organs (*n* = 12), metabolic diseases (*n* = 15), genetic syndromes that include brain dysgenesis (*n* = 5) or a combination of these (*n* = 10). None of the excluded newborns were treated with THT.

Baseline characteristics of the patients during hospitalization, e.g., UapH, base deficit and Apgar scores are shown in Table 1. Comparing term and preterm infants with and without THT the cohort showed no significant differences regarding gender, birth weight, length and head circumference as well as gestational age. Significant differences were seen regarding seizures, pathologic aEEG patterns, Apgar scores at 5 min after birth, base deficit and severity of HIE. A total of 40 (36%) infants showed a pathological aEEG. A proportion of 12% of the infants showed no or mild clinical signs of a HIE (13/108), whereas criteria of a moderate and severe HIE were met by 21/108 (19%) infants. Seizures occurred in 26% (*n* = 28), palsy in 12% (*n* = 13) and coma in 9% (*n* = 10) of the study population. A total of 39 of 108 infants have been treated with THT. Of these, 8 have been late preterm and 31 term newborns. A total of 69 infants (64%, 12 preterm and 57 term newborns) have not been treated with THT. A proportion of 13% of all preterm and 55% of all term infants showed signs of a severe HIE. The mortality rate ranged from 13% in preterm to 19% in term infants with THT. The overall mortality rate was 8% in asphyctic infants (Table 1).

### 3.2. Follow-Up Examinations

A total of 20 term infants with THT and 11 late preterm infants with THT were examined after (corrected) 24 months using the BSID 2 (Table 2). Thus, a lost-to-follow-up of 51% (*n* = 55) has to be noted. The somatic outcome at the age of (corrected) 24 months shows no difference between late preterm infants with and without THT (47.8 ± 2.5 vs. 49.2 ± 1.0 cm, *p* = 0.286, Table 2), as well as between preterm and term newborns with THT (49.2 ± 1.0 vs. 47.9 ± 1.7 cm, *p* = 0.204, Table 2). A proportion of 13% (*n* = 4/31) of all children with THT assessed with BSID 2 showed a severe and 32% (*n* = 10/31) a moderate mental and/or psychomotor developmental delay. No preterm infants with THT showed major developmental disabilities.

From a total of 11 late preterm infants, who were examined after 24 months, 7 had been treated with THT (Table 2). All of these infants had moderate (*n* = 6) to severe (*n* = 1) HIE. None of the included preterm infants showed a severe developmental delay. Two infants showed a moderate developmental delay regarding mental skills (MDI 82 and 78). Only one infant showed both MDI and PDI below average (MDI 80 and PDI 84), whereas the others showed age-appropriate results.

**Figure 2:**
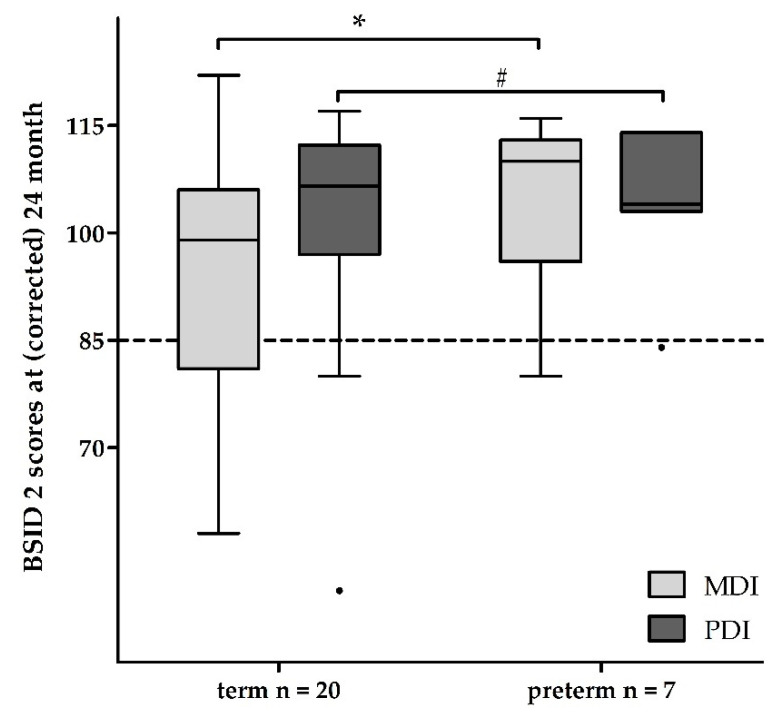
Neurodevelopmental long-term outcome after hypothermia in asphyctic term and late preterm infants at the age of (corrected) 24 months. Term and late preterm infants with hypothermia treatment have an age-appropriate development. Term outliers show developmental delays up to a score of 50, whereas preterm outliers (●) show delays up to a score of 85. Abbreviations: *n*—number, BSID 2—Bayley Scales of Infant Development 2, MDI—mental developmental index, PDI—psychomotor developmental index; * *p* < 0.05, # *p* > 0.05.

The mean MDI and PDI did not differ between preterm infants with and without THT (mean MDI 105 ± 13 with and 96 ± 20 SD without THT, *p* = 0.527; mean PDI 105 ± 11 with and 105 ± 15 SD without THT, *p* = 0.927; Table 2). In comparison, long-term outcomes of term and preterm infants with THT showed no differences regarding psychomotor development (mean PDI 101 ± 16 vs. 105 ± 11 SD, *p* = 0.570; Table 2). Preterm infants with THT had a better, age-appropriate mental development compared to term infants with THT (mean MDI 105 ± 13 vs. 93 ± 18 SD, *p* = 0.048; mean PDI 105 ± 11 vs. 101 ± 16 SD, *p* = 0.570; Table 2), as shown in Figure 2.

## 4. Discussion

In our cohort, 0.5% (108 of 20,890) of newborns showed a perinatal asphyxia with subsequent moderate to severe HIE in 0.3% (54 of 20,890). These results are in agreement with the literature: in developed countries the incidence of perinatal asphyxia amounts to 3–5 from 1000 newborns with 0.5–1 per 1000 live births affected by moderate to severe HIE [34]. 

A mortality rate of 13% (preterm) to 19% (term) in our infants with THT, respectively, and 8% in all asphyctic infants is slightly below the overall mortality rate of 30% as reported in a Cochrane meta-analysis of eleven randomized and controlled studies including 1468 infants and 436 death births [34]. A proportion of 12.5% of our included preterm infants died (1/8). Matching this, Herrera at al. [24] reported death in 18.2% (4/30) of all preterm infants with THT. There were more deaths in the group of term and late preterm infants with THT compared to the group without THT (7 vs. 2), which could be due to the fact that there were more children with severe HIEs (18 vs. 3). The main emphasis was on the comparison of preterm and term children treated with THT. Those groups showed similar baseline characteristics that make a comparison feasible.

In our study group, only 9.7% (3/31) of all survivors showed a major mental and only 3.2% (1/31) a major psychomotor developmental delay (defined as an developmental index below 70 using the BSID 2), compared to 22% (296/1344) showing a major mental and 30.1% (198/657 survivors) showing a major motor developmental delay in the already mentioned meta-analysis [34]. In conclusion, the investigated cohort in this study performed better after THT in terms of mental and psychomotor developmental delays than shown in comparable studies. The unique power of this finding lies in the characteristics of the disease profile of the participants of each study. Thus, our study should be handled carefully and should not be the only instrument in decision-making regarding THT in preterm infants. 

In our study group, one of eight late preterm infants (12.5%) showed a mild developmental delay after a moderate HIE with THT. Herrera et al. [24] addressed the short- and long-term outcome of preterm infants after THT in a retrospective analysis of 22 patients with perinatal asphyxia. Since outcomes have been assessed in different ways, they are only comparable to a limited extent. They reported 38.9% moderate to severe neurodevelopmental impairment among survivors. We report a mean MDI of 105 (80–116) and a mean PDI of 105 (84–114) using BSID 2, while Herrera et al. report a mean MDI of 84 (54–110) and a mean PDI of 83 (46–118) using BSID 3 [24]. 

To our knowledge this is the first study to report that THT in late preterm may result in age-appropriate mental and psychomotor development, compared to late preterm infants without and term infants with THT. Nevertheless, data of late preterm infants without THT are comparable only to a limited extent, as they had no or moderate HIE. With a lost-to-follow-up of 51.8% of the patients in the present study our treatment effect and outcome might be overestimated in the case that many of the missing developmental scores were not age appropriate. Nevertheless, in our subgroup of preterm infants with THT, only one infant missed the follow-up.

As the sample size of our single-center pilot study is small and given the retrospective study design, the presented results remain descriptive and should be interpreted with caution. This pilot study provides a tentative hypothesis and no conclusive statistics. Since it is a retrospective and not an experimental design, no power analysis has been performed. Therefore, these data should not be used as the basis for decisions in a clinical context.

We did not address adverse effects of THT in late preterm infants as its documentation was, due to the retrospective design, incomplete. In term infants a significant rate increment of bradycardia and thrombocytopenia could be found in hypothermia groups. Moreover, an effect of therapeutic hypothermia on the incidence of clinically recognized seizures that was on the borderline of significance has been described [34]. Furthermore, Rao et al. reported complications in 90% out of a total of 31 preterm infants who underwent THT. Hyperglycemia was more likely in preterm than in term infants [23].

## 5. Conclusions

Here we show age-appropriate mental and psychomotor development in late preterm infants with moderate HIE after THT. Merely a single preterm with THT showed a mild mental and psychomotor developmental delay at the age of (corrected) 24 months. This result highlights the importance of studying THT in asphyctic late preterm infants. Against the background of literature, these results provide the first data of asphyctic premature infants with age-appropriate mental and psychomotor development until the age of (corrected) 24 months after THT. Nevertheless, due to its limitations, particularly the sample size and lost-to-follow-up, the data may not be used for clinical decision making. Currently, the NICHD Neonatal Research Network is carrying out a randomized controlled clinical study, which assesses the safety and effectiveness of whole body hypothermia in preterm infants at 33–35 weeks gestational age (ID No.: CN-01540535) [35]. The results must be awaited before recommendations can be made for this vulnerable patient group.

## Figures and Tables

**Table 1:** Baseline characteristics of newborns with and without therapeutic hypothermia at discharge.

		Late Preterm Newborn			Term Newborn		
		with THT (*n* = 8)	without THT (*n* = 12)	*p*	with THT (*n* = 31)	without THT (*n* = 57)	*p*
**female**	N (%)	4 (50)	5 (42)	† b	15 (48)	19 (53)	† b
**gestational age [weeks]**	mv ± sd	35 ± 0.8	35 ± 0.9	† b	39.5 ± 1.2	39.3 ± 1.4	† b
**birth weight [gram]**		2681 ± 522	2504 ± 432	† b	3599 ± 576	3394 ± 600	† b
**head circumference [cm]**		34 ± 1	33 ± 2	† b	35 ± 2	35 ± 3	† b
**max. base excess [mmol/L]**		−23 ± 5	−14 ± 4	* b	−21 ± 5	−17 ± 4	* b
**umbilical cord pH**		6.9 ± 0.2	7.1 ± 0.2	* b	6.9 ± 0.2	7.0 ± 0.2	* b
**Apgar score at 5′**	median ± IQR	4 ± 3	5 ± 3	† b	4 ± 4	6 ± 3	** b
**seizures**	*n* (%)	5 (63)	0	* a	19 (61)	4 (7)	* a,c
**palsy**		3 (38)	0	* a	10 (32)	0	* a,c
**coma**		1 (13)	0	† a	9(29)	0	* a,c
**pathologic aEEG**		8 (100)	2 (17)	* a	27 (87)	3 (5)	** a
**HIE**				** a			** a
no		0	9 (75)		0	38 (67)	
mild		0	2 (17)		0	5 (9)	
moderate		7 (88)	0		14 (45)	12 (21)	
severe		1 (13)	1 (8)		17 (55)	2 (4)	
**death**		1(13)	0	† a	6 (19)	2 (4)	† a

* *p* < 0.05, ** *p* < 0.001, † *p* > 0.05, a chi-square test, b t-test, c correction of the alpha error according to Bonferroni–Holm. Abbreviations: *n*–number in total, THT–therapeutic hypothermia, max.–maximal, pH–potentia Hydrogenii, **′**–minutes, IQR–interquartile range, aEEG–amplitude-integrated electroencephalogram, HIE–hypoxic-ischemic encephalopathy, mv–mean value, sd–standard deviation.

**Table 2:** Developmental status of late preterm newborns with and without HIE, as well as term newborns with therapeutic hypothermia at the age of (corrected 24) months.

		Late Preterm Newborn			Term Newborn	
		without THT (*n* = 4)	with THT (*n* = 7)	*p*	with THT (*n* = 20)	*p*
**female**	*n* (%)	1 (25)	3 (43)	† a	9 (45)	† a
**age at birth [months]**	mv ± sd	35 ± 1	35 ± 1	† a	40 ± 1	† a
**weight [gram]**		11,435 ± 1198	12,208 ± 1116	† a	12,492 ± 1677	* a
**head circumference [cm]**		47.8 ± 2.5	49.2 ± 1.0	† a	47.9 ± 1.7	† a
**HIE**	*n* (%)					
no		2 (50)	0		0	
mild		0	0		0	
moderate		2 (50)	6 (86)	* b,d	11 (55)	† b,d
severe		0	1 (14)	† b,d	9 (45)	† b,d
**MDI**	mv ± sd	96 ± 20	105 ± 13	† c	93 ± 18	* c
≥115	*n* (%)	1 (25)	1 (14)		1 (5)	
≥85		1 (25)	5 (72)		13 (65)	
<85		2 (50)	1 (14)		3 (15)	
<70		0	0		3 (15)	
**PDI**	mv ± sd	105 ± 15	105 ± 11	† c	101 ± 16	† c
≥115	*n* (%)	1 (25)	1 (14)		1 (5)	
≥85		3 (75)	5 (72)		15 (75)	
<85		0	1 (14)		3 (15)	
<70		0	0		1 (5)	

* *p* < 0.05, † *p* > 0.05, a *t*-test, b Chi-Square test, c Mann–Whitney-U test, d correction of the alpha error according to Bonferroni-Holm. Abbreviations: *n*–number in total, max.–maximal, HIE–hypoxic-ischemic encephalopathy, mv–mean value, sd–standard deviation, MDI–mental developmental index, PDI–psychomotor developmental index.

## Data Availability

The data presented in this study are available on request from the corresponding author. The data are not publicly available due to patients’ privacy.
